# The Thyroid Twist: How GLP-1 Agonists Are Influencing Autoimmune Thyroid Care

**DOI:** 10.7759/cureus.98153

**Published:** 2025-11-30

**Authors:** Angela D Mazza

**Affiliations:** 1 Endocrinology, Metabolic Center for Wellness, Oviedo, USA

**Keywords:** autoimmune thyroid disease, glp-1 receptor agonists, graves’ disease, hashimoto thyroiditis, immunometabolism, obesity, semaglutide, thyroid autoimmunity, tirzepatide

## Abstract

Autoimmune thyroid disease (AITD), including Hashimoto thyroiditis and Graves’ disease, represents the most prevalent organ-specific autoimmunity and a major cause of thyroid dysfunction worldwide. Increasingly, AITD coexists with metabolic disorders such as obesity, insulin resistance, and metabolic dysfunction-associated steatotic liver disease (MASLD), suggesting an interplay between immune dysregulation and metabolic health. Glucagon-like peptide-1 receptor agonists (GLP-1RAs), such as semaglutide and tirzepatide, have emerged as transformative agents for type 2 diabetes and obesity through their potent effects on weight reduction, insulin sensitivity, and cardiovascular outcomes. Beyond these metabolic benefits, accumulating evidence indicates that GLP-1RAs exert immunomodulatory effects, including suppression of pro-inflammatory cytokines, enhancement of regulatory T-cell function, and improvements in adipose tissue and gut-derived immune signaling.

Although dedicated trials of GLP-1RAs in AITD are lacking, preliminary reports suggest potential impact on thyroid volume, thyroid function, and autoimmune activity. Mechanistic hypotheses include reduced leptin-driven T-helper (Th)1/Th17 activity, improved adipokine balance, and modulation of gut-thyroid axis pathways. Potential benefits must be weighed against safety considerations, including theoretical risks of thyroid C-cell tumors from animal studies and the need for careful adjustment of thyroid hormone therapy in patients experiencing significant weight loss.

This review synthesizes the current understanding of AITD pathophysiology, summarizes emerging evidence on the immunometabolic effects of GLP-1RAs, and explores potential therapeutic implications for patients with concurrent thyroid autoimmunity and metabolic disease. Rigorous clinical and translational studies are needed to clarify the role of GLP-1RAs in the management of AITD.

## Introduction and background

Introduction

Autoimmune thyroid disease (AITD), encompassing Hashimoto thyroiditis and Graves’ disease, is the most common organ-specific autoimmune disorder, affecting up to 5% of the general population, with a marked female predominance of nearly 5:1 compared with men [1,2]. AITD frequently coexists with features of metabolic syndrome, including obesity, insulin resistance, dyslipidemia, and metabolic dysfunction-associated steatotic liver disease (MASLD), reflecting the bidirectional interplay between immune dysregulation and metabolic stress [3,4]. Obesity and type 2 diabetes (T2D) are themselves associated with higher rates of thyroid dysfunction and altered autoimmunity, underscoring the convergence of metabolic and endocrine pathways [5,6]. In parallel, glucagon-like peptide-1 receptor agonists (GLP-1RAs), such as semaglutide and tirzepatide, have transformed the therapeutic landscape of T2D and obesity by producing profound weight reduction, improvements in glycemic control, and cardiovascular risk reduction [7,8]. Beyond their metabolic benefits, GLP-1RAs demonstrate immunomodulatory effects, including attenuation of systemic inflammation and modulation of immune cell activity, raising the possibility of relevance to autoimmune conditions such as AITD [9]. To date, however, the intersection of GLP-1RA therapy and AITD remains underexplored.

Methodology

This narrative review was performed by a structured search of PubMed, Embase, and Scopus from database inception through August 2025. Search terms included combinations of autoimmune thyroid disease, Hashimoto thyroiditis, Graves’ disease, thyroid autoimmunity, GLP-1 receptor agonist, semaglutide, tirzepatide, incretin therapy, immunometabolism, and metabolic inflammation. To enhance coverage across endocrinology, obesity, diabetes, and immunology, key specialty journals were also reviewed, and reference lists of relevant articles were hand-searched.

The electronic searches identified approximately 410 records. After removal of duplicates, 290 unique records remained for title and abstract screening. Of these, 160 articles were excluded because they did not address thyroid autoimmunity, thyroid outcomes, GLP-1RAs, or relevant immunometabolic pathways. The remaining 130 full-text articles were assessed for relevance. Studies were included if they: 1) Addressed autoimmune thyroid disease (Hashimoto thyroiditis, Graves’ disease, or related thyroid autoimmunity); 2) Evaluated GLP-1RAs and reported thyroid, hepatic-metabolic, or immune/inflammatory outcomes; or 3) Provided mechanistic or integrative background on adipokines, immunometabolism, gut microbiota, or inflammatory pathways relevant to both GLP-1 biology and AITD.

Articles were excluded at full-text review if they: 1) Lacked thyroid-specific or immunometabolic outcomes; 2) Focused solely on diabetes/obesity management without relevance to autoimmunity, immunometabolism, or thyroid function; or 3) Represented duplicate data, commentaries, or editorials without additional primary or mechanistic content.

A total of 68 articles were included in the final synthesis (corresponding to the reference list), comprising clinical trials and observational studies of GLP-1RA, mechanistic and immunology papers on GLP-1 signaling and metabolic inflammation, literature on autoimmune thyroid disease pathophysiology, adipokines, Non-alcoholic fatty liver disease (NAFLD)/Metabolic dysfunction-associated fatty liver disease (MAFLD)/Metabolic dysfunction-associated steatotic liver disease (MASLD)/Metabolic dysfunction-associated steatohepatitis (MASH), and gut-thyroid-microbiota interactions. Given the narrative design of this review, inclusion was based on conceptual and mechanistic relevance rather than rigid methodological scoring or quantitative pooling.

## Review

Pathophysiology of AITD

Hashimoto Thyroiditis

Hashimoto thyroiditis is characterized by a breakdown of immune tolerance to thyroid autoantigens, primarily thyroid peroxidase (TPO) and thyroglobulin (Tg), leading to lymphocytic infiltration of the thyroid gland and progressive tissue damage [1,10]. The immunopathogenesis is driven by a predominance of T helper (Th)1-mediated immune responses, with elevated interferon-γ (IFN-γ) production and impaired function of regulatory T cells (Tregs), which normally restrain autoreactive lymphocytes [11,12]. Over time, this chronic immune attack results in a gradual loss of functional thyroid tissue, with the clinical course typically progressing from an initial euthyroid state to subclinical hypothyroidism and, in a subset of patients, overt hypothyroidism requiring lifelong thyroid hormone replacement [13,14]. Environmental triggers such as excess iodine intake, viral infections, and psychosocial stress may accelerate disease onset in genetically predisposed individuals [15]. The heterogeneity of progression underscores the complex interplay between immune dysregulation, thyroid reserve, and environmental influences in determining clinical outcomes.

Graves’ Disease

Graves’ disease is an AITD driven by the production of thyroid stimulating hormone (TSH) receptor antibodies (TRAb), which aberrantly stimulate the TSH receptor (TSHR) and result in thyroid hormone overproduction and hyperthyroidism [2,16]. The immune response in Graves’ disease is characterized by involvement of both Th2 and Th17 pathways, with interleukin (IL)-4, IL-6, and IL-17 contributing to B-cell activation and antibody production, while defects in immune checkpoints, such as impaired regulatory T-cell activity, promote loss of tolerance [17,18]. Beyond thyroidal hyperfunction, Graves’ disease may manifest with extra-thyroidal complications, most notably thyroid-associated orbitopathy, in which fibroblasts and preadipocytes in the orbit express the TSHR and insulin-like growth factor 1 receptor (IGF-1R), leading to tissue expansion, edema, and fibrosis under autoantibody stimulation [19,20]. Less commonly, patients develop pretibial dermopathy and acropachy, reflecting fibroblast activation in the skin and extremities [21]. Together, these features illustrate Graves’ disease as a systemic autoimmune condition with complex immune signaling pathways that extend beyond the thyroid gland.

GLP-1RAs: Beyond glycemic control

GLP-1RAs such as semaglutide and the dual glucose-dependent insulinotropic polypeptide (GIP)/GLP-1RAs tirzepatide enhance glucose-dependent insulin secretion, suppress glucagon, slow gastric emptying, and reduce appetite through central pathways. Tirzepatide’s additional activation of the GIP receptor further amplifies its insulinotropic and metabolic effects. GLP-1RAs mimic the incretin hormone GLP-1 to enhance glucose-dependent insulin secretion, suppress glucagon release, delay gastric emptying, and reduce appetite through central nervous system pathways [21,22]. These mechanisms account for their robust effects on glycemic control and weight reduction in patients with T2D and obesity. Beyond pancreatic actions, GLP-1RAs exert direct effects in the hypothalamus and brainstem to regulate satiety and food intake, as well as gastrointestinal effects that slow nutrient absorption, contributing to postprandial glucose control [23,24].

In addition to metabolic regulation, GLP-1RAs exhibit anti-inflammatory and immunomodulatory properties. Experimental and clinical studies have demonstrated reductions in systemic inflammatory markers such as C-reactive protein, IL-6, and tumor necrosis factor (TNF)-α [9,25]. GLP-1 signaling has been shown to inhibit activation of the NLRP3 inflammasome, a key driver of innate immune-mediated inflammation, and to enhance Treg activity, suggesting a broader role in restoring immune tolerance [26,27]. These pathways position GLP-1RAs as potential modulators of autoimmunity by attenuating chronic low-grade inflammation and shifting immune balance toward regulation. Furthermore, GLP-1RA-induced weight loss alters adipokine profiles, lowering leptin and increasing adiponectin, which may reduce Th1/Th17 polarization and improve insulin sensitivity, thereby indirectly influencing autoimmune disease activity [28].

**Figure 1:**
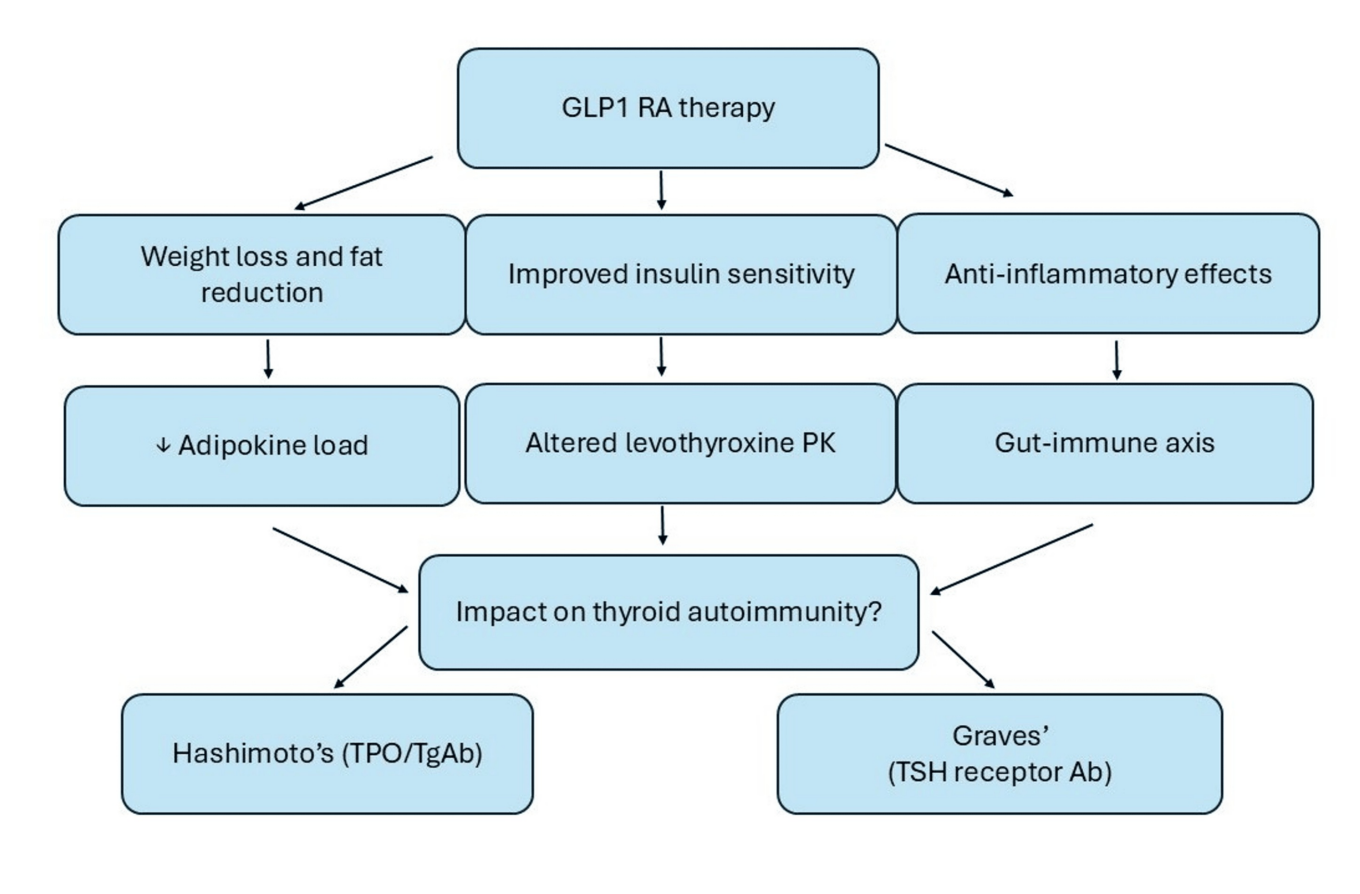
Proposed theoretical mechanisms linking GLP-1RAs and autoimmune thyroid disease (AITD) GLP-1RA: Glucagon-like peptide-1 receptor agonists; PK: pharmacokinetics; TPO: thyroid peroxidase antibody; TgAb: thyroglobulin antibody; TSH receptor Ab: thyroid stimulating hormone (TSH) receptor antibody These pathways are hypothesis-generating only, as current evidence is limited. GLP-1RAs may potentially influence AITD indirectly through weight loss, improved insulin sensitivity, anti-inflammatory effects, and gut–immune interactions. These physiologic shifts could alter thyroid hormone dynamics and immune activity, but definitive causal links have not been established. Image credit: Created by Mazza AD using Microsoft Powerpoint (Microsoft Corp., Redmond, WA, USA).

The impact of GLP-1RAs on thyroid physiology remains incompletely understood. Case reports and observational data have noted reductions in thyroid volume and TSH levels in obese individuals treated with GLP-1RAs though the clinical significance is unclear [29]. Concerns about thyroid safety largely stem from rodent studies, where chronic GLP-1RA exposure was associated with C-cell hyperplasia and medullary thyroid carcinoma, findings not confirmed in human trials [30,31]. Occasional reports describe thyroid nodules or altered thyroid function tests in patients on GLP-1RAs, but there is little direct evidence linking these agents to thyroid autoimmunity [32]. Thus, while thyroid safety remains under surveillance, the broader evidence suggests that the benefits of GLP-1RAs extend beyond glucose lowering to encompass immunometabolic effects that could plausibly influence AITD (Figure 1).

Potential mechanisms of interaction between GLP-1RAs and AITD

Weight Loss and Adipose Tissue Immunology

Adipose tissue is increasingly recognized as an active immunologic organ that contributes to systemic inflammation and autoimmunity through secretion of adipokines and pro-inflammatory cytokines [33]. In obesity, elevated leptin levels promote Th1 and Th17 polarization, impair regulatory T-cell function, and enhance autoreactive immune responses, while hypoadiponectinemia removes a key anti-inflammatory brake [34,35]. Weight loss induced by lifestyle interventions or GLP-1RAs reduces circulating leptin and increases adiponectin, shifting the immune balance toward greater Treg activity and reduced Th1/Th17 responses [36,37]. These changes may attenuate the inflammatory milieu that fuels AITD, in which adipose-derived cytokines such as TNF-α, IL-6, and IL-17 are known to exacerbate the thyroidal immune attack [11,38]. Moreover, adipose tissue macrophages in obesity adopt a pro-inflammatory M1 phenotype, further amplifying systemic immune activation, whereas weight reduction favors a switch toward an anti-inflammatory M2 phenotype [39]. By reducing the inflammatory load of adipose tissue, weight loss and adipokine normalization may therefore represent an indirect but clinically meaningful mechanism by which GLP-1RAs influence thyroid autoimmunity.

Gut-Thyroid Axis and Microbiome Modulation

The gut microbiome plays a critical role in shaping systemic immune responses, and alterations in microbial composition have been implicated in the development of AITD [40,41]. Increased intestinal permeability (“leaky gut”) allows microbial products such as lipopolysaccharides to trigger systemic inflammation and loss of self-tolerance, thereby amplifying autoimmune processes [42]. GLP-1RAs have been shown to improve gut barrier integrity by enhancing tight junction protein expression and reducing endotoxemia, thereby lowering systemic inflammatory burden [23,42]. In addition, GLP-1RA therapy has been associated with favorable shifts in gut microbiota composition, including enrichment of short-chain fatty acid-producing bacteria, which promote Treg activity and suppress pro-inflammatory Th17 responses [43,44]. These changes may attenuate systemic autoimmunity and indirectly influence thyroid-specific immune responses. Early studies suggest that modulation of the gut-thyroid axis through GLP-1RAs represents a plausible mechanism linking metabolic therapies to improved autoimmune outcomes, though dedicated studies in AITD are needed [45,46].

Direct Thyroidal Effects?

Evidence for direct actions of GLP-1RAs on thyroid tissue remains limited and controversial. Early preclinical studies suggested GLP-1 receptor expression in thyroid C cells, forming the basis for rodent findings of C-cell hyperplasia and medullary thyroid carcinoma under chronic GLP-1RA exposure [47,48]. However, subsequent human studies have demonstrated minimal or absent GLP-1 receptor expression in thyroid follicular cells and inconsistent detection in parafollicular C cells, raising doubt about the translational relevance of rodent models [49,50]. Clinical trial data and post-marketing surveillance have not confirmed an increased incidence of medullary thyroid carcinoma in GLP-1RA-treated patients, though long-term monitoring continues [30,31]. Beyond potential direct receptor-mediated effects, it is more plausible that GLP-1RAs influence thyroid physiology indirectly by modulating systemic inflammation, adipokine signaling, and immune tolerance, thereby affecting the autoimmune milieu rather than acting on thyroid tissue itself [9]. This distinction underscores the importance of viewing GLP-1RAs as systemic immunometabolic modulators with secondary effects on thyroid autoimmunity, rather than as agents with direct thyroidal targets.

Metabolic-Immune Crosstalk

Metabolic dysfunction, particularly insulin resistance and MASLD, amplifies systemic inflammation and contributes to autoimmune disease activity [50,51]. Hyperinsulinemia and ectopic lipid accumulation promote activation of pro-inflammatory pathways, including Nuclear Factor kappa-light-chain-enhancer of activated B cells (NF-κB) signaling and NLRP3 inflammasome activation, which in turn exacerbate loss of immune tolerance [52,53]. In the context of AITD, insulin resistance has been associated with higher titers of thyroid autoantibodies and more rapid progression to overt hypothyroidism [5]. GLP-1RAs improve insulin sensitivity, reduce hepatic steatosis, and attenuate systemic inflammatory mediators, thereby targeting upstream drivers of autoimmunity [54,55]. Clinical and experimental studies demonstrate that GLP-1RAs decrease hepatic fat content and fibrosis markers, reduce circulating pro-inflammatory cytokines, and promote favorable adipokine profiles, collectively dampening immune activation [9,25]. By modulating this immunometabolic axis, GLP-1RAs may indirectly attenuate thyroid autoimmunity, supporting their potential role as dual-purpose agents in patients with coexisting metabolic and AITD.

Clinical Evidence to Date

To date, no randomized controlled trials (RCTs) have specifically evaluated the impact of GLP-1RAs on AITD. The majority of available evidence comes from case reports, small observational studies, and secondary analyses of large clinical trials. Some studies in obese and diabetic cohorts have demonstrated reductions in thyroid volume and TSH levels following treatment with exenatide or liraglutide, suggesting possible effects on thyroid morphology and function [29,56,57]. These findings are generally attributed to weight loss and improved metabolic status rather than direct thyroidal effects, although the underlying mechanisms remain unclear.

Isolated case reports and small series have described altered thyroid function tests in patients treated with GLP-1RAs, including suppressed TSH or fluctuating free thyroid hormone levels, but these changes were typically transient and not associated with progression to overt thyroid dysfunction [30,32]. Importantly, the pivotal cardiovascular and obesity outcome trials of GLP-1RAs, including the Semaglutide Unabated Sustainability in Treatment of Type 2 Diabetes (SUSTAIN), Semaglutide Treatment Effect in People with obesity (STEP), and SURPASS (tirzepatide) programs, did not report clinically meaningful increases in AITD or sustained thyroid dysfunction [7,8,30]. Post-marketing surveillance has similarly not identified a strong signal for increased thyroid autoimmunity, though vigilance persists given preclinical findings in rodent C cells.

Emerging retrospective real-world analyses have suggested a possible association between GLP-1RA use and lower incidence of hypothyroidism, particularly with semaglutide; however, these observations remain preliminary and require prospective confirmation [58,59]. Importantly, subclinical hypothyroidism can represent an adaptive, obesity-related metabolic response, and weight loss itself is known to improve inflammatory and immune profiles. Thus, any apparent thyroid-related benefits seen with GLP-1RAs may reflect weight-loss-mediated changes rather than a drug-specific effect. This underscores the need for well-designed trials capable of distinguishing GLP-1RA-specific immune or thyroid effects from those attributable to weight reduction alone. At present, evidence remains hypothesis-generating, and dedicated mechanistic and disease-specific studies are needed to determine whether GLP-1RAs have clinically meaningful impact on the incidence or progression of AITD.

Potential Risks and Safety Considerations

The principal thyroid-related safety concern with GLP-1RAs arises from rodent studies demonstrating C-cell hyperplasia and medullary thyroid carcinoma (MTC) following chronic exposure [47,60]. These findings are attributed to GLP-1 receptor expression in rodent thyroid C cells, which differs significantly from humans, where receptor expression is sparse and inconsistent [48,61]. Large cardiovascular outcome trials (e.g., Liraglutide Effect and Action in Diabetes: Evaluation of Cardiovascular Outcome Results (LEADER), SUSTAIN) and post-marketing surveillance have not demonstrated an increased incidence of MTC in humans, though long-term pharmacovigilance continues given the rarity of this malignancy [30,62]. Consequently, GLP-1RAs remain contraindicated in individuals with a personal or family history of MTC or multiple endocrine neoplasia type 2 (MEN2), despite the absence of definitive human risk.

Another limitation is the lack of long-term data specifically addressing AITD outcomes in patients receiving GLP-1RAs. While preliminary studies and observational signals suggest potential thyroidal effects, no RCTs have evaluated progression of Hashimoto thyroiditis or Graves’ disease under GLP-1RA therapy [32,58]. Without disease-specific endpoints, it is uncertain whether immunomodulatory and metabolic benefits translate into clinically meaningful alterations in thyroid autoimmunity. Furthermore, most of the available evidence comes from secondary analyses of diabetes or obesity cohorts, which may not be fully generalizable to patients with established AITD.

In patients with Graves’ disease or hypothyroidism requiring levothyroxine, significant GLP-1RA-induced weight loss may alter thyroid hormone pharmacokinetics and necessitate medication dose adjustments [63]. Weight reduction decreases lean and fat mass, influencing thyroid hormone distribution, and may lower levothyroxine requirements [64]. Similarly, improved metabolic clearance may impact antithyroid drug dosing in Graves’ disease. Although not contraindicated, clinicians should closely monitor thyroid function tests and adjust therapy as weight and metabolic status evolve under GLP-1RA treatment [65]. This emphasizes the need for proactive endocrine follow-up when GLP-1RAs are prescribed to patients with concomitant autoimmune thyroid disorders.

Future directions

Rigorous mechanistic and clinical studies are needed to clarify whether GLP-1RAs modify the course of AITD. Foundational work should include translational trials that pair GLP-1RA therapy with deep immunophenotyping (Th1/Th17/Treg balance, inflammasome activity), adipokine profiling (leptin, adiponectin), and gut-barrier/microbiome readouts, alongside thyroid-specific endpoints (Thyroid Peroxidase Antibodies (TPOAb)/Thyroglobulin Antibodies (TgAb)/TRAb trajectories, ultrasound echogenicity and volume, Doppler vascularity, and patient-reported outcomes) [1,3,9,22]. Parallel randomized studies in Hashimoto thyroiditis and Graves’ disease should pre-specify metabolic co-endpoints (Homeostatic Model Assessment for Insulin Resistance (HOMA-IR) and Magnetic Resonance Imaging-Proton Density Fat Fraction (MRI-PDFF) for liver fat) to test the immunometabolic hypothesis suggested by observational signals and secondary analyses [32,60].

**Figure 2:**
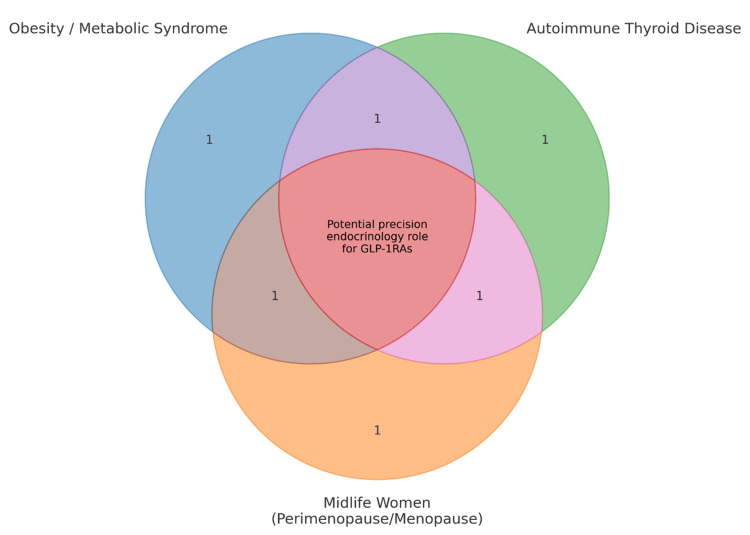
Conceptual rationale for studying GLP-1RAs in autoimmune thyroid disease (AITD) This schematic illustrates why patients with AITD often share clinical features with populations already treated using Glucagon-like peptide-1 receptor agonist (GLP-1RAs)—such as obesity, insulin resistance, Metabolic Dysfunction-Associated Steatotic Liver Disease (MASLD), and midlife hormonal transition. These overlapping factors contribute to heightened inflammatory and metabolic burden, raising the hypothesis that GLP-1RAs might offer benefit through weight loss, improved metabolic health, or reduced inflammation. The figure highlights the theoretical, biochemical, and clinical rationale, while emphasizing that these connections are not proven and require targeted clinical trials. Image credit: Created by Mazza AD using Microsoft Powerpoint (Microsoft Corp., Redmond, WA, USA).

From a precision-endocrinology perspective, GLP-1RAs could serve dual aims in patients who harbor both metabolic dysfunction (obesity/insulin resistance/MASLD) and thyroid autoimmunity-attenuating inflammatory tone while improving weight, glycemia, and hepatic steatosis [7,50,54]. Given the strong female predominance of AITD and the midlife rise in metabolic risk during the menopausal transition, targeted trials in perimenopausal and postmenopausal women are warranted, with attention to hormone status, body-composition change, cardiovascular risk, and dose adjustments of thyroid medications during weight loss [1,2,66] (Figure 2). 

Such designs could determine whether immunometabolic improvements translate into slower progression from subclinical to overt hypothyroidism or reduced relapse in Graves’ disease.

Combination strategies also merit exploration. In Graves’ disease, where extrathyroidal manifestations are mediated by TSHR/IGF-1R-expressing fibroblasts, studies could test GLP-1RAs as adjuncts to antithyroid drugs or biologics (for example, alongside IGF-1R blockade for orbitopathy) to reduce systemic inflammation and metabolic drivers without compromising disease control [19,25,67]. Platform or factorial trials could compare GLP-1RA-augmented regimens versus standard care on thyroid autoantibody kinetics, relapse rates, and steroid/antithyroid dose exposure. Throughout, safety monitoring should include thyroid function (to adjust levothyroxine/antithyroid dosing as the weight changes), calcitonin surveillance in high-risk genotypes, and standardized adverse-event adjudication.

## Conclusions

GLP-1RAs have emerged as transformative therapies in T2D and obesity, but their impact may extend beyond metabolic regulation into the realm of immune modulation. By reducing systemic inflammation, improving adipokine balance, and restoring gut barrier function, GLP-1RAs have the potential to influence autoimmune pathways relevant to thyroid disease. These pleiotropic actions suggest that GLP-1RAs could become valuable tools in the integrative management of patients with AITD, particularly those who also struggle with obesity, insulin resistance, or metabolic dysfunction.

At present, however, the evidence supporting a role for GLP-1RAs in modifying thyroid autoimmunity remains preliminary. Observational studies, case reports, and indirect mechanistic data generate compelling hypotheses but fall short of demonstrating causality or consistent clinical benefit in AITD. Large cardiovascular and obesity trials have provided reassurance regarding thyroid safety, but have not been designed to evaluate autoimmune-specific endpoints. Thus, current knowledge is largely hypothesis-generating and highlights the urgent need for well-designed clinical and translational studies.

For clinicians, awareness of both the promise and the limitations of current evidence is essential. GLP-1RAs may offer dual advantages in patients with AITD and concurrent metabolic disease. Still, careful monitoring of thyroid function and medication requirements remains critical, especially in the context of significant weight loss. Until dedicated trials are available, the use of GLP-1RAs in AITD should be viewed as an intriguing but unproven strategy, one that underscores the evolving interface between metabolism, immunity, and endocrine care.
